# Reconstruction of a left main coronary artery aneurysm using a radial artery patch

**DOI:** 10.1093/jscr/rjae423

**Published:** 2024-06-21

**Authors:** Hazel Doronila, Alan Kypson, Lance Landvater, Curtis Anderson

**Affiliations:** Division of Cardiac Surgery, Rex Cardiac Surgical Specialists, UNC Health Rex, 2800 Blue Ridge Rd, Suite 201, Raleigh, NC 27518, United States; Division of Cardiac Surgery, Rex Cardiac Surgical Specialists, UNC Health Rex, 2800 Blue Ridge Rd, Suite 201, Raleigh, NC 27518, United States; Division of Cardiac Surgery, Rex Cardiac Surgical Specialists, UNC Health Rex, 2800 Blue Ridge Rd, Suite 201, Raleigh, NC 27518, United States; Division of Cardiac Surgery, Rex Cardiac Surgical Specialists, UNC Health Rex, 2800 Blue Ridge Rd, Suite 201, Raleigh, NC 27518, United States

**Keywords:** left main coronary artery, aneurysm, radial artery patch, cardiac surgery

## Abstract

A left main coronary artery aneurysm is a rare anomaly. There are no standardized treatment guidelines given the infrequency of reported cases. A 70-year-old African American female with an enlarging distal left main coronary artery aneurysm was taken to the operating room for surgical intervention. The patient underwent a successful open surgical repair of the aneurysm with reconstruction of the distal left main using a radial artery patch. No coronary bypasses were necessary. Aneurysm ligation with concomitant coronary artery bypass grafting is commonly reported but reconstruction may be preferable when the anatomy is suitable. Preservation of nonobstructed native coronary artery circulation should also be considered to avoid life-long graft dependency.

## Introduction

A coronary artery aneurysm (CAA) is described as an abnormal dilatation of a coronary artery segment. The prevalence of CAAs ranges from 0.3% to 0.53% [1] of the population who undergo left heart catheterization. Left main coronary artery aneurysms are the least common, found in 0.1% of those patients who undergo cardiac catheterization [2]. The optimal treatment of CAAs remains controversial. Given the rarity of cases, recommendations are derived from small case series and anecdotal evidence. As a result, there is no standardized treatment guideline.

Treatment options include medical management with surveillance imaging, and percutaneous or surgical intervention. Surgical intervention is commonly chosen when there is associated fistula formation, external mechanical compression, rupture, need for concomitant cardiac surgery, or involvement of multiple coronary arteries, a giant (>20 mm, or > 4× reference vessel) coronary artery aneurysm or a left main coronary artery (LMCA) [3].

Open surgical repair aims to exclude the aneurysm(s) and reestablish flow. Reported techniques vary and include aneurysm ligation with bypass grafting, resection with reconstruction, or marsupialization with interposition graft. LMCA aneurysms located near the left main trunk orifice and behind the main pulmonary artery present a challenge in aneurysm ligation. Some case studies reported temporary transection of the main pulmonary artery to assess the aneurysm [4, 5]. Extensive dissection between the aorta and pulmonary artery may be necessary [1]. Most case studies that describe surgical repair report aneurysm ligation with bypass to a distal target. Our case introduces the use of a radial artery patch to reconstruct the affected coronary artery thereby preserving native coronary artery circulation.

## Case presentation

**Figure 1:**
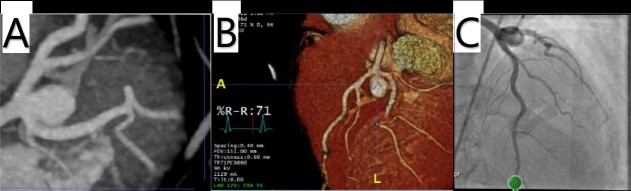
(A, B) Cardiac CTA showing a saccular aneurysm measuring 12.5 mm in length from the LMCA bifurcation to the apex of the aneurysm, and 9.04 × 10.5 mm in cross-sectional area. (C) LHC showing a distal LMCA aneurysm between the LAD and left circumflex.

A 70-year-old African American female with past medical history significant for Type II diabetes, hypertension and breast cancer was referred for a LMCA aneurysm. The aneurysm was incidentally discovered on a computed tomography (CT) scan in 2013 measuring 6 mm in diameter by 8 mm in length. Conservative management with anticoagulation was well tolerated, but follow-up CT angiography in 2021 demonstrated progressive enlargement to 10 mm by 12 mm (Fig. 1A and B). Left heart catheterization (LHC) demonstrated a saccular aneurysm extending from the distal LMCA into the origins of the left anterior descending (LAD) and circumflex arteries (Fig. 1C). Surface echocardiography showed preserved ventricular function and no valvular pathology. Due to its persistent growth, surgical repair was recommended.

**Figure 2:**
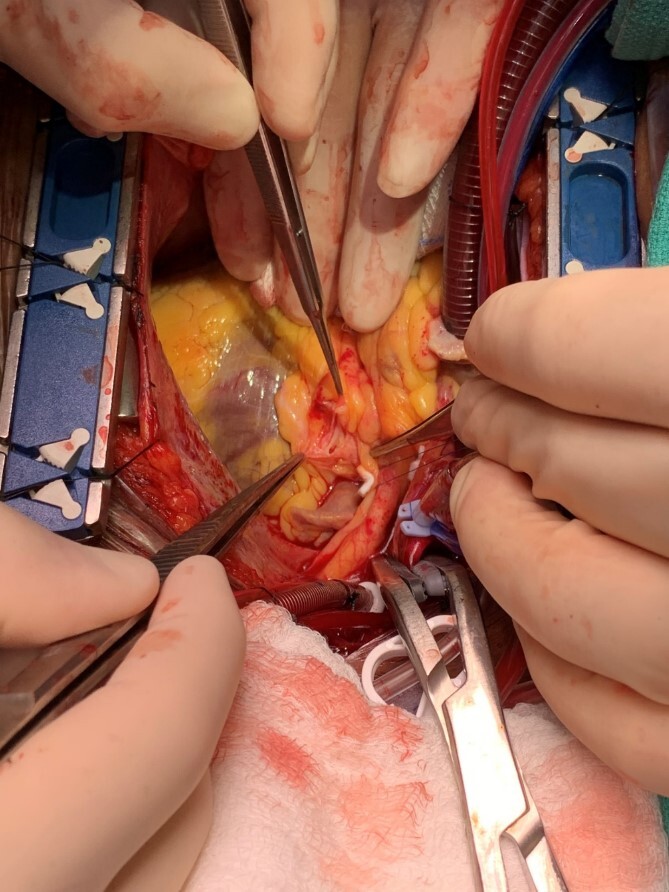
Intraoperative photo demonstrating the lumen of the distal left main coronary artery where it bifurcates into the circumflex and left anterior descending artery through the incised superior wall of the aneurysm.

**Figure 3:**
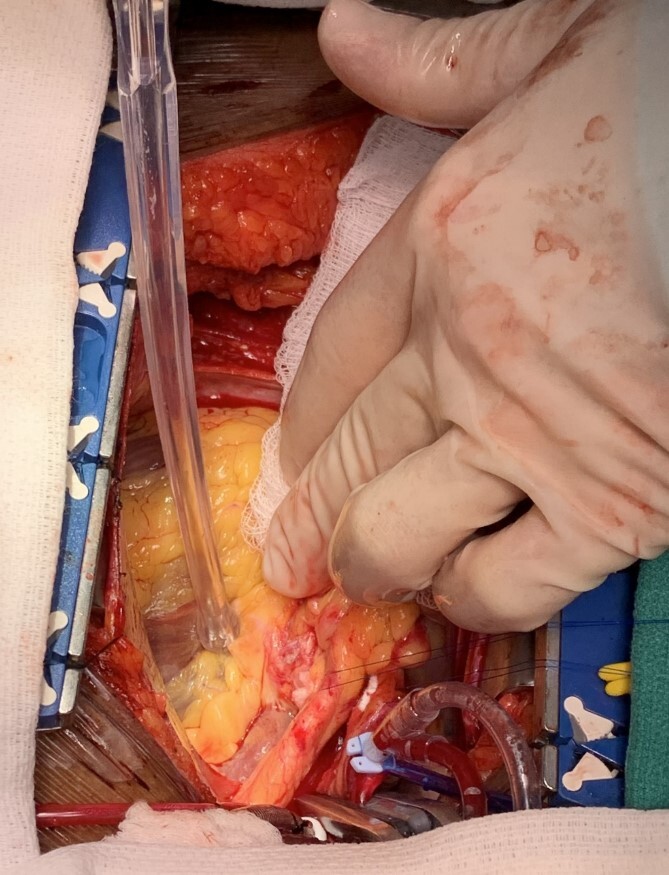
Intraoperative photo demonstrating the patch repair of the left main coronary artery aneurysm.

The patient was taken to the operating room and underwent a median sternotomy. The left radial and left internal mammary arteries (LIMA) were harvested in anticipation of LMCA ligation and bypass to the left circumflex and LAD. She was placed on cardiopulmonary bypass via standard central cannulation and the heart was arrested with antegrade and retrograde Del Nido cardioplegia. Although we were prepared to divide the pulmonary artery to provide for better exposure, we found it unnecessary. Medial retraction of the pulmonary artery provided adequate exposure of the distal LMCA. The aneurysm sac was opened and the orifices of the left main, circumflex and LAD were identified (Fig. 2). There was no thrombus within the aneurysm. There was a 10 mm by 12 mm oval aneurysmal defect with a distinct transition between normal arterial wall and the significantly attenuated aneurysmal segment. The aneurysmal defect was excised and a patch of the radial artery was used to reconstruct the superior wall of the LMCA (Fig. 3). No coronary bypasses were done. The patient separated from cardiopulmonary bypass easily. Transesophageal echocardiogram showed normal left and right ventricular function.

## Results

**Figure 4:**
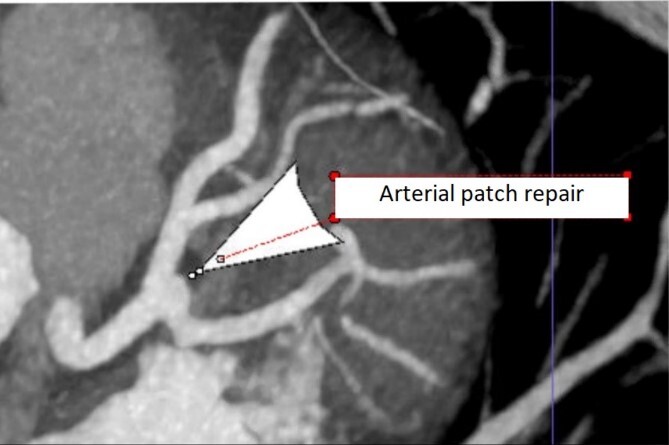
Repeat coronary CTA at 3 month follow up showing normal appearing distal LMCA.

The patient’s postoperative course was unremarkable. She was discharged home on post-operative day 6 on low dose aspirin and clopidogrel. Follow up CT angiography was performed at 3 months showing a normal appearing LMCA (Fig. 4). We recommended life-long dual anti-platelet therapy with aspirin and clopidogrel. She will be followed in our aortic surveillance clinic every 1–2 years for noninvasive imaging.

## Discussion

Although many case reports describe extensive dissection, temporary transection of the main pulmonary artery, and aneurysm ligation with bypass to a distal target, our patient’s anatomy allowed for an overall less invasive surgical approach.

We obtained adequate exposure of the LMCA aneurysm and surrounding vasculature with medial retraction of the main pulmonary artery so temporary ligation was not necessary. Our initial operative plan was to ligate the LMCA and bypass the LAD and circumflex arteries using two arterial conduits (the LIMA and radial artery). However, the borders of the aneurysm were so distinct from the normal arterial wall that we elected to reconstruct the LMCA instead. This patient’s LMCA aneurysm was located at the bifurcation of the LAD and circumflex arteries. When the dome of the aneurysm was opened, we could clearly visualize the orifices of the circumflex and LAD. We could discern a distinct transition between normal arterial wall and the attenuated tissue comprising the wall of the saccular aneurysm. The normal arterial segments had good tissue integrity and average caliber that held sutures well in contrast to the aneurysmal segment which was notably thin-walled, friable, and clearly dilated. Therefore, the abnormal tissue was excised and the LMCA was reconstructed. The radial artery that had been harvested was utilized with the thought that an arterial patch would be most appropriate to withstand arterial pressures. Moreover, since the ostia of the LAD and circumflex were patent and the patient had no obstructive coronary artery disease found on LHC, no bypass grafting was done.

Had the LMCA been ligated and bypassed, the patient would have become completely graft dependent. Resection with patch-graft reconstruction maintains native coronary circulation without concern for long-term graft patency. Margins were not sent for confirmation as the discrete transition from attenuated aneurysmal tissue to normal tissue made us confident in our resection. The potential for recurrent aneurysm formation was discussed but given the tissue integrity of the remaining wall, we perceived this to be less risk than life-long graft dependency. Periodic non-invasive imaging will be pursued to ensure the continued integrity of the repair and to evaluate for aneurysm recurrence.
